# A cross‐species post‐translational modification profiling of histones by LC‐MS/MS revealed conserved oxidative modifications in plants

**DOI:** 10.1111/nph.71354

**Published:** 2026-06-22

**Authors:** Sau‐Shan Cheng, Wai‐Ching Sin, Yuen‐Yi Chau, Hon‐Ming Lam

**Affiliations:** ^1^ School of Life Sciences, Science Centre The Chinese University of Hong Kong Shatin New Territories, Hong Kong (SAR) China; ^2^ Centre for Soybean Research of the State Key Laboratory of Agrobiotechnology The Chinese University of Hong Kong Shatin New Territories, Hong Kong (SAR) China

**Keywords:** abiotic stress, crops, oxidation and hydroxylation, plant epigenetics, PTM peptidoform

## Abstract

Conservation of post‐translational modifications (PTMs) in histones across six plant species.

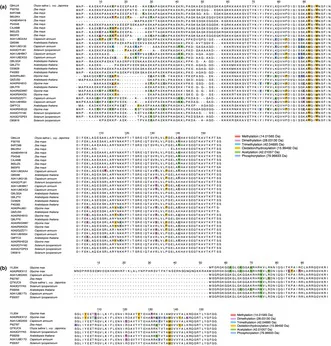

## Disclaimer

The New Phytologist Foundation remains neutral with regard to jurisdictional claims in maps and in any institutional affiliations.

## Introduction

Plants are constantly exposed to environmental stresses that induce extensive reprogramming of gene expression, driving the transcription and translation of stress‐responsive factors. At the heart of this adaptive response lie histone proteins, whose N‐terminal tails are dynamically modified by post‐translational modifications (PTMs) (Kouzarides, 2007) – including acetylation, phosphorylation, methylation, ubiquitination, and ADP‐ribosylation (Bannister & Kouzarides, 2011). These chemical marks form a critical epigenetic layer that modulates gene expression without altering the DNA sequence, enabling rapid, reversible adaptations to environmental cues (Bannister & Kouzarides, 2011). While lysine acetylation and methylation have been thoroughly characterized in mammalian systems, the landscape of other histone PTMs – particularly in plants – remains largely uncharted.

Histone PTMs and their genomic localization have traditionally been mapped using antibody‐based techniques such as chromatin immunoprecipitation followed by sequencing (ChIP‐seq), which can link specific marks to *cis*‐regulatory elements and provide spatial contexts inaccessible by mass spectrometry (MS) alone (Lu *et al*., 2021). However, antibody‐dependent methods are inherently limited by epitope accessibility and the availability of modification‐specific antibodies: a significant constraint given the combinatorial complexity and dynamic nature of histone PTMs, which often preclude discrimination between distinct modifications at the same residue or identical marks on similar sequence motifs (Lu *et al*., 2021; Yao *et al*., 2024). Although emerging nanopore‐based technologies offer promise for direct PTM mapping (Chang *et al*., 2025), their application to histones is complicated by heterogeneous charge states in complex protein samples (Rukes & Cao, 2025). As a result, MS‐based approaches remain the most unbiased, high‐throughput, and comprehensive platform for the simultaneous identification and quantification of multiple PTMs (Lu *et al*., 2021; Rukes & Cao, 2025). Recent advances in MS have begun to uncover novel stress‐responsive histone PTMs; however, systematic, quantitative profiling of these marks across plant species – and the degree to which they are conserved – remains critically lacking.

In this study, we demonstrated that our LC‐MS/MS‐based workflow enables a robust, high‐resolution platform for the comprehensive profiling of histone PTMs across six agronomically vital plant species, encompassing both monocots (*Zea mays* and *Oryza sativa*) and dicots (*Glycine max*, *Capsicum annuum*, *Solanum lycopersicum*, and *Arabidopsis thaliana*). We reported the discovery of previously undocumented oxidation and hydroxylation patterns on core histones (H1–H4) in plants. By quantitatively tracking the conserved histone peptidoforms, we pinpointed the potential stress‐responsive remodeling within three distinct histone regions under salt, drought, and heat stress in rice and soybean. These findings not only expand the plant histone code to include dynamic oxidative modifications but also reveal their condition‐specific plasticity, highlighting their potential roles in chromatin reorganization and abiotic stress adaptations.

## Results

### Conservation of histone PTM patterns across diverse plant species

To address this gap and assess the conservation of histone PTM patterns across the plant kingdom, we enriched the nuclear proteins from six agronomically important species representing both monocotyledonous and dicotyledonous lineages. These included *Zea mays* ‘Tasty Sweet F1’ (corn) and *Oryza sativa* L. ssp. *japonica* (rice), as representatives of monocots, and *Glycine max* (L.) Merr. cv Williams 82 (soybean), *Capsicum annuum* ‘Piccante di Cayenna’ (cayenne pepper), *Solanum lycopersicum* ‘Moneymaker’ (tomato), and *A. thaliana* Col‐0, as representatives of dicots. This experimental design enabled a cross‐lineage comparison of PTM conservation, with a strategic emphasis on crops of global agricultural relevance.

Nuclear proteins were isolated from nuclei‐enriched fractions, subjected to proteolytic digestion with trypsin, and analyzed by LC‐MS/MS. Enrichment of nuclear proteins relative to total proteome extracts was confirmed by comparing their relative abundances derived from label‐free quantification (Supporting Information Fig. S1). Subcellular localization predictions using DeepLoc 2.0 further validated the robust enrichment of histone proteins in the nuclear fractions relative to total protein extracts (Fig. S2). The effective enrichment of both histone and non‐histone nuclear proteins underscores the broad applicability of our sample preparation method for nuclear proteome profiling and the LC‐MS/MS workflow across both monocot and dicot species.

**Fig. 1:**
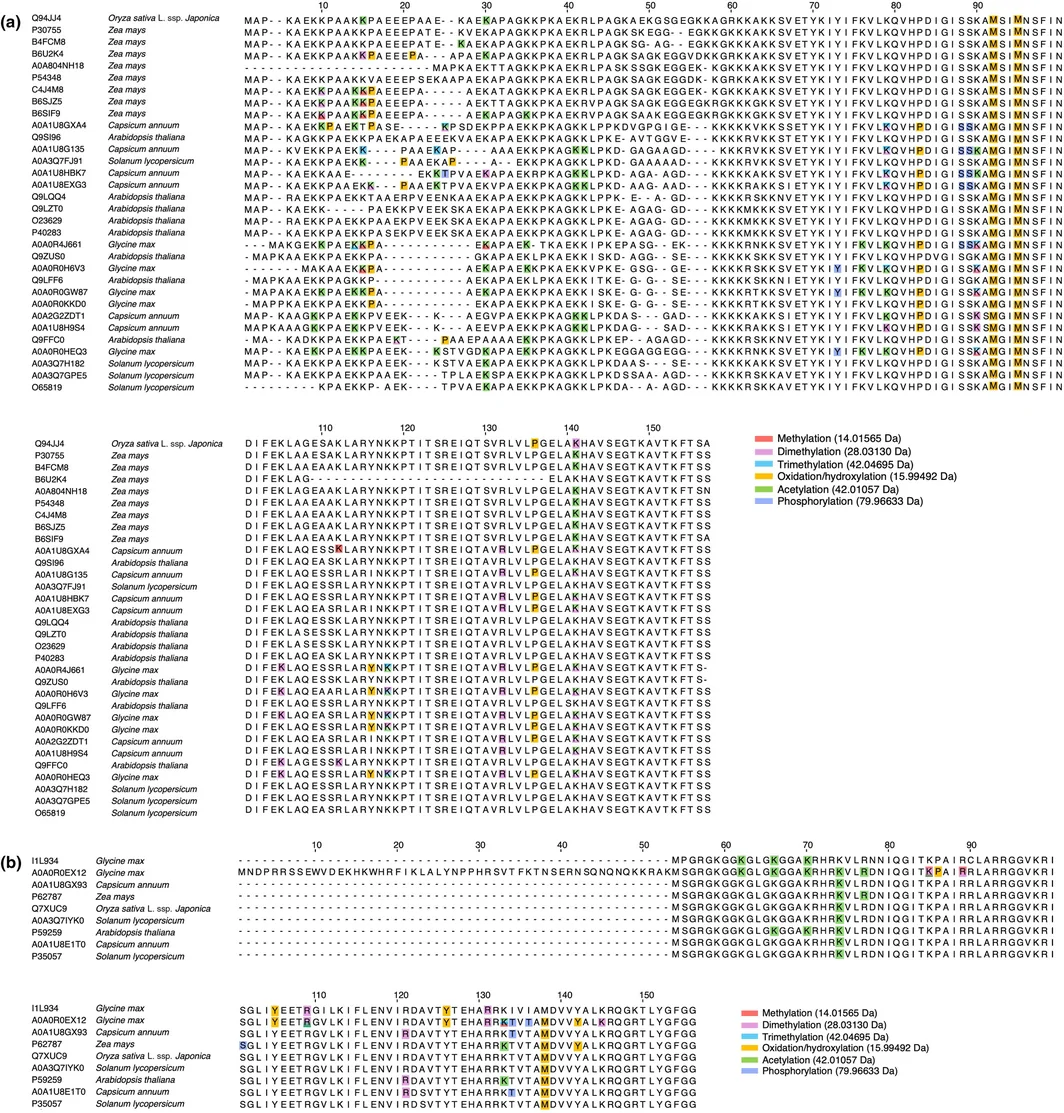
Conservation of post‐translational modifications (PTMs) in histones across six plant species. Multiple sequence alignment of histone H2B (a) and H4 (b) orthologs identified by LC‐MS/MS from *Zea mays*, *Oryza sativa*, *Glycine max*, *Capsicum annuum*, *Solanum lycopersicum*, and *Arabidopsis thaliana*. Sequences were aligned using Clustal Omega (Sievers & Higgins, 2018). Modified residues and their corresponding PTMs are color‐coded as follows: red, methylation (+14.01565 Da); magenta, dimethylation (+28.03130 Da); cyan, trimethylation (+42.04695 Da); yellow, oxidation/hydroxylation (+15.99492 Da); green, acetylation (+42.01057 Da); purple, phosphorylation (+79.96633 Da). Alignments for histones H1, H2A, and H3 are shown in Supporting Information Figs S3–S5.

We identified tryptic peptides corresponding to linker histone H1 and core histones H2A, H2B, H3, and H4, with an emphasis on detecting histone peptidoforms bearing acetylation (+42.01057 Da), methylation (+14.01565 Da), dimethylation (+28.03130 Da), trimethylation (+42.04695 Da), phosphorylation (+79.96633 Da), and oxidation or hydroxylation (+15.99492 Da) marks. To investigate the conservation and divergence of PTMs among orthologous histones, we generated multiple sequence alignments of identified histone proteins from the species included in our study. These alignments revealed shared modification hotspots as well as lineage‐specific patterns based on detected peptidoforms. Representative alignments for histones H2B and H4 are shown in Fig. 1; alignments for H1, H2A, and H3 are provided in Figs S3–S5.

### Site‐specific oxidative modifications on core histones revealed by LC‐MS/MS

Our nuclear enrichment and LC‐MS/MS workflow successfully captured several previously reported PTMs, including acetylation at H4K20, H3K18, and H3K23. These marks were consistently detected across histone orthologs from all six plant species examined, particularly in H2B, H3, and H4, suggesting the possibility of their evolutionary conservation (Kaimori *et al*., 2016; Klein *et al*., 2019; Wang *et al*., 2021). When comparing the PTMs observed on H2B, H3, and H4 with previously documented plant PTMs (Willems *et al*., 2024), our results aligned well with the established modification patterns in plants, indicating that several histone residues might serve as conserved sites of modification (Table S1). Furthermore, we noted distinct combinations of modified and unmodified residues within individual histone peptides, underscoring the sensitivity and specificity of our streamlined approach for comprehensive PTM profiling (Figs S6, S7). Notably, we identified a mass shift of +15.99492 Da – which corresponds to either oxidation on methionine residues (resulting in methionine sulfoxide formation) or hydroxylation on proline or tyrosine residues across multiple histones. To our knowledge, these site‐specific oxidative modifications have not been previously documented in the plant species investigated (Figs 1, S2–S4).

As an example of site‐specific PTM detection, we analyzed the MS/MS spectra of a conserved tryptic peptide from histone H4 (KVLRDNIQGITKPAIR), which served as a potential acetylation site at H4K20 (H4K20ac) – a modification mark previously reported in Arabidopsis (Zhang *et al*., 2007) and mammalian systems (Kaimori *et al*., 2016). Our results confirmed the presence of this modification in all six plant species studied, demonstrating the effectiveness of our extraction protocol and LC‐MS/MS workflow across both monocots and dicots (Fig. S6a). In addition to this well‐characterized modification, we detected the H4 peptide TVTAMDVVYALKR, which carries methionine oxidation at position 84 (H4M84ox), in both monocot and dicot species (Fig. S6b).

### Histone oxidation correlates with abiotic stress responses in rice and soybean

To assess whether the relative abundances of these PTMs are influenced by abiotic stress, we quantified the H4K20ac and H4M84ox levels under various treatments in rice (*O. sativa*) and soybean (*G. max*), which represent staple monocot and dicot crops that are frequently exposed to abiotic stress (Pagano & Miransari, 2016; Sackey *et al*., 2025). Global proteomic changes observed in soybean under salt, heat, and drought stress, and in rice under salt stress, confirmed physiological stress responses in these plants (Figs S8–S11). Comparative analyses revealed elevated abundances of the H4K20ac‐bearing peptide (K_Ac_VLRDNIQGITKPAIR) and the H4M84ox‐bearing peptide (TVTAM_Ox_DVVYALKR) under salt stress in both species (Fig. S6c,d,g,h). By contrast, neither modified peptide showed significant changes in heat‐stressed soybean (Fig. S6f,j), whereas TVTAM_Ox_DVVYALKR showed reduced abundance under drought in soybean (Fig. S6i). Together, these patterns suggest that these histone PTMs might respond in a stress‐specific manner.

**Fig. 2:**
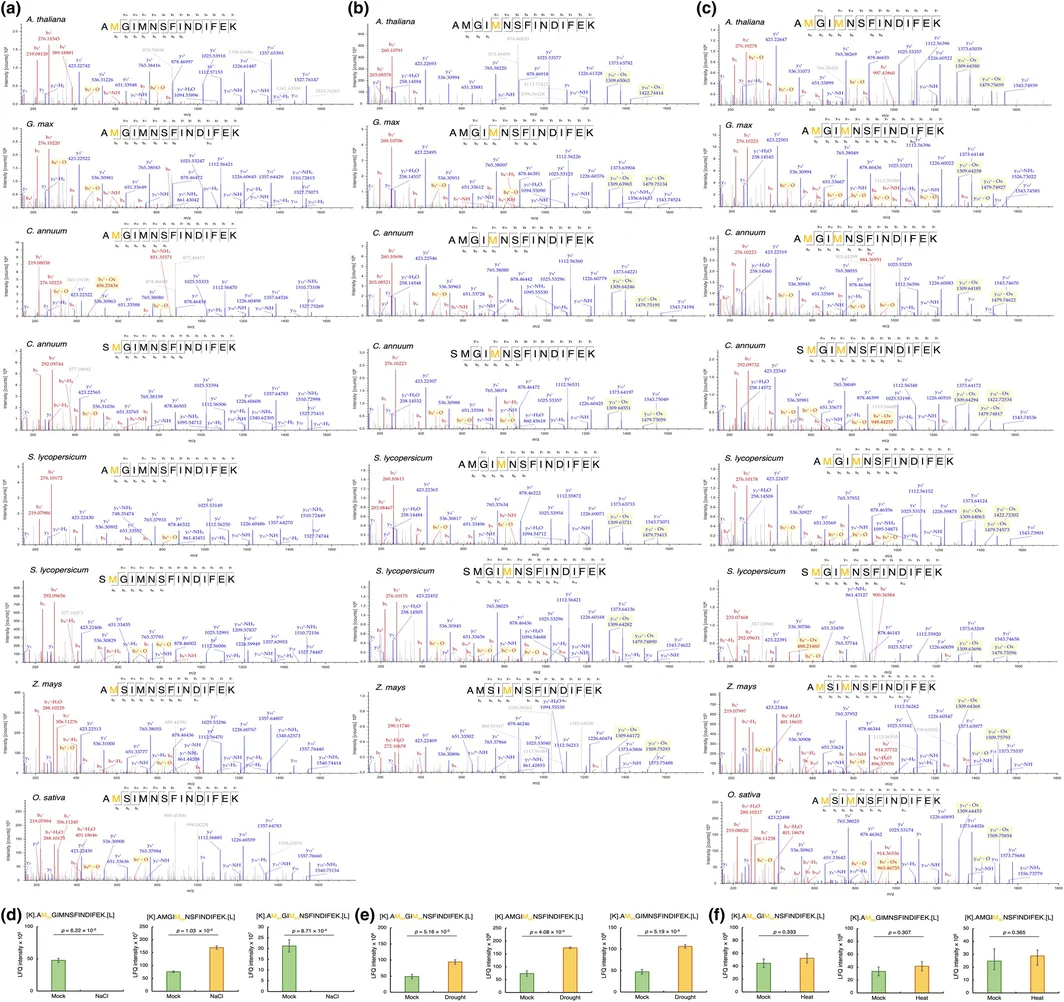
Conserved histone H2B methionine oxidation peptidoforms and their stress‐specific remodeling. (a–c) Representative LC‐MS/MS spectra confirming multiple oxidative post‐translational modifications (PTMs) on a conserved histone H2B ortholog peptide (A/SMGIMSFINDFEK) in *Zea mays*, *Oryza sativa*, *Glycine max*, *Capsicum annuum*, *Solanum lycopersicum*, and *Arabidopsis thaliana*. Spectra correspond to the peptidoforms A/SM_ox_GIMSFINDFEK (a), A/SMGIM_ox_SFINDFEK (b), and A/SM_ox_GIM_ox_SFINDFEK (c) with oxidized methionine residues boxed in yellow on the annotated b/y‐ion series. (d–f) Relative abundances of oxidized peptidoforms in *G. max* under salt (d), drought (e), and heat (f) stress conditions. Data represent mean ± SE (*n* = 3 biological replicates), determined by label‐free quantification and analyzed by one‐tailed Student's *t*‐test. The MS/MS spectra of the corresponding unmodified peptides are provided in Supporting Information Fig. S12.

During sample preparation for MS analysis, unintended oxidation can occur at methionine, cysteine, and other susceptible residues due to prolonged exposure to oxygen, potentially confounding the accurate assignment of endogenous histone oxidation and hydroxylation marks (Chang *et al*., 2025). Conversely, residues such as cysteine, methionine, and tyrosine are also physiological targets of regulatory oxidative modifications, which have emerged as a potential link between cellular redox status and metabolism‐driven epigenetic control (Gantner *et al*., 2024). To distinguish between spurious oxidation introduced during sample handling and biologically meaningful oxidative PTMs (Chang *et al*., 2025), we interrogated a conserved histone H2B peptide ortholog containing multiple oxidized methionine residues across all plant species (Fig. 2a–c). We then assessed whether the modification remained consistent across different stress conditions.

We identified four distinct tryptic peptidoforms of this conserved H2B sequence – A/SM_ox_GIM_ox_SFINDFEK, A/SM_ox_GIMSFINDFEK, A/SMGIM_ox_SFINDFEK, and the unmodified form, A/SMGIMSFINDFEK – across all six species examined. Their presence in both monocots and dicots suggests that oxidative modifications at the orthologous H2B M74 and M77 positions may be evolutionarily conserved (Figs 2a–c, S12). Notably, these combinatorial PTM variants displayed pronounced shifts in relative abundances under specific abiotic stress conditions, revealing dynamic remodeling tailored to distinct environmental cues (Fig. 2d–f). In salt‐treated soybean, AMGIM_ox_SFINDFEK emerged as the predominant – and sole detectable – oxidized peptidoform (Fig. 2d). By contrast, drought stress induced elevated levels of all three oxidized variants, including the doubly oxidized form AMGIM_ox_SFINDFEK (Fig. 2e), whereas heat stress elicited no significant change in any peptidoform (Fig. 2f). These stress‐specific patterns demonstrate that oxidation at these sites is not stochastic but dynamically regulated, pointing to a functional role in environmental adaptations.

## Discussion

Histone modifications are central to the regulation of gene expression and genome functions, shaping chromatin landscapes that govern transcription, DNA replication, and repair (Zhang *et al*., 2003). Depending on the residue and modification type, these PTMs can activate or repress target genes. For instance, acetylation and phosphorylation can alter histone charge, modulating chromatin accessibility and transcriptional output through electrostatic mechanisms (Strahl & Allis, 2000; Zhang *et al*., 2003). Beyond classical modifications, histones are increasingly recognized as sensors of the cellular redox state. Oxidation of redox‐sensitive residues – particularly methionine, cysteine, and tyrosine – can induce structural perturbations, including partial nucleosome unfolding and thermal destabilization, thereby fine‐tuning the chromatin architecture and gene expression under oxidative stress (Gantner *et al*., 2024; Palma *et al*., 2024).

Our streamlined workflow enabled the isolation of nuclear proteins while preserving a comprehensive global pattern of PTMs with high reproducibility across species, offering a powerful tool to accelerate plant nuclear proteome studies. Here, we applied this LC‐MS/MS‐based approach to profile histone PTMs across six phylogenetically diverse species, encompassing both monocots and dicots. We confirmed the high sequence conservation of core histones H1–H4 and the preservation of numerous known PTM sites (Figs 1, S3–S5, Table S1). Crucially, our method was able to resolve complex combinatorial PTMs, including coexisting modifications on distinct residues and alternative states on the same residue, as exemplified by the multi‐PTM peptidoforms on histone orthologs (Fig. S13).

Leveraging this resolution, we focused on a + 15.99492‐Da mass shift on methionine – a hallmark of oxidation potentially linked to oxidative stress – that exhibited both conserved positioning across species and stress‐dependent remodeling. Although oxidation‐related PTMs, such as H3 glutathionylation, have been documented in mammalian histones (García‐Giménez *et al*., 2013), specific methionine oxidation or related oxidative PTMs have not been reported in plant histones in peer‐reviewed studies. The consistent, site‐specific oxidation marks across monocots and dicots suggest these modifications might be evolutionarily conserved, and their potential functional relevance could extend beyond the model systems.

Salt, drought, and heat stress are major abiotic challenges that induce reactive oxygen species (ROS) accumulation (Kerchev & Van Breusegem, 2022). Yet they differ in primary damage mechanisms, subcellular ROS sources, and downstream signaling pathways in different species (Krasensky & Jonak, 2012). These variations might contribute to the stress‐specific remodeling of oxidative histone PTMs observed in this study. Using rice and soybean as representative monocot and dicot species, respectively, we investigated whether the relative abundances of histone PTMs changed under different stress conditions (Figs 2, S6). While our study focused on oxidation and hydroxylation events (+15.99492 Da), histones are also susceptible to other oxidative modifications – including carbonylation, nitration, and disulfide bonding – that were not analyzed in this study (Gantner *et al*., 2024). Although the biological significance of distinct combinations of histone modifications has not been fully resolved, our LC‐MS/MS workflow demonstrated sufficient sensitivity to detect combinatorial PTMs across diverse species (Figs 1, S3–S5, S13). While MS‐based approaches cannot unambiguously distinguish hydroxylation from oxidation (Verrastro *et al*., 2015), our comparative analyses might provide experimental evidence that oxidative PTMs represent a previously underappreciated aspect of the plant histone code. Furthermore, the observed changes in peptidoform abundance under salt, drought, and heat stress imply that these modifications might play a role in stress‐responsive chromatin regulation. Future experiments utilizing mutants that lack specific PTM sites or modifications, along with ChIP–seq analyses, will be essential to determine whether these modifications are linked to particular genomic loci and to clarify their roles in chromatin reconfiguration, gene regulation, and stress adaptations.

## Materials and Methods

### Plant materials and growth conditions

*Capsicum annuum* ‘Piccante di Cayenna’, *Zea mays* ‘Tasty Sweet F1’, and *Solanum lycopersicum* ‘Moneymaker’ seeds were germinated in medium‐grade vermiculite for 7–10 d and subsequently transplanted into Floragard potting soil. Plants were grown until the V1 stage (when primary leaves were fully expanded) under standard glasshouse conditions. Wild‐type *A. thaliana* (Col‐0) seeds were surface‐sterilized and sown on half‐strength Murashige and Skoog (½MS) agar medium. Seedlings were maintained in a growth chamber at 22–24°C, 70–80% relative humidity, 80–120 μE m^−2^ s^−1^ light intensity, with a 16 h : 8 h light : dark photoperiod for 11 d before harvest. Seeds of *Glycine max* (L.) Merr. cv Williams 82 (soybean) were germinated in medium‐grade vermiculite for 7 d and then transferred to a hydroponic system containing ½‐strength Hoagland's nutrient solution.

Soybean growth and salinity treatment followed previously described protocols (Hoagland & Arnon, 1950; Liu *et al*., 2019). Briefly, at the V1 stage, plants were transferred to ½‐strength Hoagland's solution supplemented with 0.9% (w/v) NaCl for 1 d, while control plants remained in NaCl‐free solution. Drought stress was induced at the V1 stage by transferring plants to half‐strength Hoagland's solution containing 200 mM mannitol for 1 d.

For heat treatments, soybean seedlings were germinated in vermiculite and grown in plant growth chambers (GEN1000‐GE; Conviron, Winnipeg, MB, Canada) under a 16 h : 8 h light : dark photoperiod at 28°C/23°C, respectively, with a light intensity of 400 μmol m^−2^ s^−1^. At the V1 stage, plants were exposed to 38°C under a 16 h : 8 h light : dark photoperiod for 1 d, whereas control plants were maintained at 16 h : 8 h light : dark, 28°C : 23°C.

For *Oryza sativa* L. ssp. *japonica* cv Nipponbare seeds were surface‐disinfected with 1.4% (v/v) sodium hypochlorite for 15 min and rinsed three times with distilled water. Seedlings were grown hydroponically and exposed to salt treatment following previously published procedures (Liu *et al*., 2024) with these modifications: in sucrose‐free ½MS with or without 0.5% (w/v) NaCl. Unless otherwise specified, all plant samples were harvested at the V1 developmental stage, immediately frozen in liquid nitrogen, and stored at −80°C until nuclei isolation and protein extraction.

### Nucleus enrichment and protein extraction

Nucleus enrichment from all six plant species (*Z. mays*, *O. sativa*, *G. max*, *C. annuum*, *S. lycopersicum*, and *A. thaliana*) was performed following a previously described protocol with modifications (Sikorskaite *et al*., 2013). Briefly, *c*. 2 g fresh weight of frozen plant tissue was ground to a fine powder in liquid nitrogen and resuspended in 10 volumes of nuclear isolation buffer (10 mM MES‐KOH (pH 5.4), 10 mM NaCl, 10 mM KCl, 2.5 mM EDTA, 250 mM sucrose, 0.1 mM spermine, 0.5 mM spermidine, 1 mM DTT, 1 mM phenylmethylsulfonyl fluoride (PMSF), and 1× Halt™ Protease Inhibitor Cocktail (Cat#78430; Thermo Fisher Scientific, Waltham, MA, USA)). Triton X‐100 was added to a final concentration of 0.5% (v/v) to permeabilize organelles for 20 min. The homogenate was filtered through two layers of Miracloth and centrifuged through a discontinuous density gradient consisting of 60% (v/v) Percoll (Cat#P7828; Sigma‐Aldrich, St. Louis, MO, USA) overlaid on 2.5 M sucrose at 3500 g for 30 min at 4°C. Total proteome and nucleus‐enriched protein fractions were extracted using TRIzol™ Reagent (Cat#15596018; Thermo Fisher Scientific) according to the manufacturer's protocol.

For MS sample preparation, the protein concentration was determined using the Pierce™ BCA Protein Assay Kit (Cat#23225; Thermo Fisher Scientific). Ten micrograms of protein were treated with 5 mM DTT at 37°C for 30 min, followed by 20 mM iodoacetamide at room temperature for 30 min and then 5 mM DTT at 37°C for 30 min. Pierce™ Trypsin Protease (Cat#90057; Thermo Fisher Scientific) was added at one‐twentieth (w/w) of the protein amount according to the manufacturer's instructions for digestion at 37°C overnight. The digested peptides were desalted with a Pierce™ C18 Spin Column (Cat#89873; Thermo Fisher Scientific) for later analyses.

### Liquid chromatography–tandem mass spectrometry (LC‐MS/MS) analysis

LC‐MS/MS proteomic analysis was conducted using a Fusion Lumos Tribrid Orbitrap mass spectrometer (Thermo Fisher Scientific) coupled online to an Ultimate 3000 nano‐UHPLC system (Thermo Fisher Scientific). Peptides were separated on an Acclaim PepMap RSLC 75 μm × 25 cm, C18 column (Cat#164941; Thermo Fisher Scientific). Mobile phase buffer A consisted of 0.1% (v/v) formic acid (FA) in water, and buffer B consisted of 0.1% (v/v) FA in 80% acetonitrile. Approximately 800 ng desalted peptides per sample, reconstituted with buffer A, were loaded and separated using the following 75‐min gradient at 300 nL min^−1^: 0% B (0–5 min), 0–6% B (5–8 min), 6–18% B (8–48 min), 18–30% B (48–58 min), 30–80% B (58–60 min), 80% B hold (60–65 min), 80–0% B (65–66 min), and 0% B re‐equilibration (66–75 min).

Data‐dependent acquisition was performed with a 3‐s cycle time. Full MS1 survey scans were acquired at 60 000 resolution (at *m/z* 200) over 375–1500 *m/z*, with an automatic gain control (AGC) target of 100%, maximum injection time of 50 ms, and charge state filtering from 2^+^ to 7^+^. Precursors were isolated using a 1.6 *m/z* window and fragmented by higher‐energy collisional dissociation with normalized collision energy of 30%. MS2 spectra were acquired at 15 000 resolution (at *m/z* 200), with an AGC target of 100%, a maximum injection time of 250 ms, and a dynamic exclusion of 40 s.

### Mass spectrometry data analysis

Raw LC‐MS/MS files were processed using Proteome Discoverer (PD) v.2.4 (Thermo Fisher Scientific) with species‐specific proteome databases: *A. thaliana* (TAIR10), *C. annuum* (Proteome ID: UP000222542), *Z. mays* (Proteome ID: UP000007305), *S. lycopersicum* (Proteome ID: UP000004994), *O. sativa* L. ssp. *japonica* cv Nipponbare (Proteome ID: UP000059680), and *G. max* (Gmax_508_Wm82.a4.v1). Database searches utilized the built‐in SEQUEST‐HT (Thermo Fisher Scientific) search engine with a Percolator‐based false discovery rate validation at 1% (*q*‐value ≤ 0.01) for both protein and peptide spectrum matches.

Tryptic peptides were searched, allowing up to two missed cleavages. Precursor and fragment mass tolerances were set to 10 ppm and 0.02 Da, respectively. Relative proteins/peptides quantification was performed in PD using Feature Mapper and Precursor Ion Quantifier nodes with default parameters. For total proteome and nuclear/total comparisons, the dynamic modifications included methionine oxidation (+15.99492 Da) and N‐terminal acetylation/Met‐loss (+26.01521 Da), with static cysteine carbamidomethylation (+57.02146 Da). In addition, nuclear histone PTM analyses included these dynamic peptide modifications: oxidation/hydroxylation (+15.99492 Da; M/P/Y), mono‐/di‐/tri‐methylation (+14.01565/+28.03130/+42.04695 Da; K/R), acetylation (+42.01011 Da; K/R), and phosphorylation (+79.96633 Da; S/T/Y). PTM localization was scored via IMP‐ptmRS, and Peptide Isoform Grouper was enabled for peptidoform‐specific quantification. In brief, peptide sequences were matched to the theoretical b‐ and y‐ion masses, and PTM sites were reported only when the corresponding site‐determining b‐ or y‐ions were observed. Subcellular predictions employed DeepLoc‐2.0 (Thumuluri *et al*., 2022). Histone peptidoform alignments were generated with Clustal Omega (Sievers & Higgins, 2018). Conserved PTM patterns across plant species were validated with the Plant PTM Viewer 2.0 (Willems *et al*., 2024). In short, a representative histone ortholog from each plant species was used to BLAST search against the PTM database in Plant PTM Viewer 2.0, and only those statistically significant matches with PTM matched site >1 were included.

## Competing interests

None declared.

## Author contributions

H‐ML designed the research strategy. S‐SC and W‐CS conducted the experiments. S‐SC, W‐CS and Y‐YC handled all the plant materials and treatments, and analyzed the data. S‐SC and W‐CS wrote the first draft of the manuscript. S‐SC, W‐CS, and H‐ML finalized the manuscript. H‐ML acquired the funding. All authors have read and agreed to the published version of the article.

## Supporting information


**Fig. S1** Principal component analysis of proteome profiles demonstrating successful nuclear protein enrichment.
**Fig. S2** Volcano plots of differential protein abundances confirming nuclear enrichment.
**Fig. S3** Conservation of histone H1 post‐translational modifications across five plant species.
**Fig. S4** Conservation of histone H2A post‐translational modifications across six plant species.
**Fig. S5** Conservation of histone H3 post‐translational modifications across six plant species.
**Fig. S6** Site‐specific histone H4 post‐translational modifications and their dynamic remodeling in response to abiotic stress.
**Fig. S7** Representative MS/MS spectra of selected unmodified peptides from histone H4.
**Fig. S8** Enriched gene ontology terms of the differentially expressed proteins in *O. sativa* upon salt treatment.
**Fig. S9** Enriched gene ontology terms of the differentially expressed proteins in *G. max* upon salt treatment.
**Fig. S10** Enriched gene ontology terms of the differentially expressed proteins in *G. max* upon drought treatment.
**Fig. S11** Enriched gene ontology terms of the differentially expressed proteins in *G. max* upon heat treatment.
**Fig. S12** Representative MS/MS spectra of selected unmodified peptides from histone H2B.
**Fig. S13** Combinatorial histone post‐translational modifications (PTMs) on conserved histone H2B and H3 peptides.
**Table S1** Comparison of conserved histone PTMs with reported PTM patterns in Plant PTM Viewer 2.0.
Please note: Wiley is not responsible for the content or functionality of any Supporting Information supplied by the authors. Any queries (other than missing material) should be directed to the *New Phytologist* Central Office.

## Data Availability

The mass spectrometry data from this publication have been deposited in the PRIDE database via ProteomeXchange and assigned the identifier PXD074734 (https://www.ebi.ac.uk/pride/archive/projects/PXD074734).
