# Supplementary material for: A cross‐species post‐translational modification profiling of histones by LC‐MS/MS revealed conserved oxidative modifications in plants
Source: New Phytol. 2026 Jun 22;252(2):538–45. doi: 10.1111/nph.71354 (PMC13583417; doi:10.1111/nph.71354)
Supplement: Supplementary file 1 [file NPH-252-538-s001.docx]

## *New Phytologist* Supporting Information

Article title: **A cross-species post-translational modification profiling of histones by LC-MS/MS revealed conserved oxidative modifications in plants**

Authors: Sau-Shan Cheng^1,2^,Wai-Ching Sin^1,2^, Yuen-Yi Chau^1,2^, Hon-Ming Lam^1,2*^

Article acceptance date: 30 May 2026

The following Supporting Information is available for this article:

**Fig. S1.** Principal component analysis (PCA) of proteome profiles demonstrating successful nuclear protein enrichment.

**Fig. S2.** Volcano plots of differential protein abundances confirming nuclear enrichment.

**Fig. S3.** Conservation of histone H1 post-translational modifications (PTMs) across five plant species.

**Fig. S4.** Conservation of histone H2A post-translational modifications (PTMs) across six plant species.

**Fig. S5.** Conservation of histone H3 post-translational modifications (PTMs) across six plant species.

**Fig. S6.** Site-specific histone H4 post-translational modifications (PTMs) and their dynamic remodeling in response to abiotic stress.

**Fig. S7.** Representative MS/MS spectra of selected unmodified peptides from histone H4.

**Fig. S8.** Enriched gene ontology (GO) terms of the differentially expressed proteins (DEPs) in *O. sativa* upon salt treatment.

**Fig. S9.** Enriched gene ontology (GO) terms of the differentially expressed proteins (DEPs) in *G. max* upon salt treatment.

**Fig. S10.** Enriched gene ontology (GO) terms of the differentially expressed proteins (DEPs) in *G. max* upon drought treatment.

**Fig. S11.** Enriched gene ontology (GO) terms of the differentially expressed proteins (DEPs) in *G. max* upon heat treatment.

**Fig. S12.** Representative MS/MS spectra of selected unmodified peptides from histone H2B.

**Fig. S13.** Combinatorial histone post-translational modifications (PTMs) on conserved histone H2B and H3 peptides.

**Table S1.** Comparison of conserved histone PTMs with reported PTM patterns in Plant PTM Viewer 2.0.

**Fig. S1.** **Principal component analysis (PCA) of proteome profiles demonstrating successful nuclear protein enrichment**. The plots visualize label-free quantification-based protein abundances from nucleus-enriched fractions versus total proteome extracts across all samples from *Z. mays*, *O. sativa*, *G. max*, *C. annuum*, *S. lycopersicum*, and *A. thaliana*. Data were generated using the scatterplot3d R package with default parameters (Ligges *et al.*, 2003), where each point represents an individual biological replicate (n = 3).

**Fig. S2. Volcano plots of differential protein abundances confirming nuclear enrichment.** Volcano plots depicting the differential protein abundances between nucleus-enriched fractions and total proteome extracts in *Z. mays*, *O. sativa*, *G. max*, *C. annuum*, *S. lycopersicum*, and *A. thaliana*. Proteins detected in all biological replicates (n = 3) of either fraction were analyzed for differential abundance using edgeR (Robinson *et al.*, 2009; McCarthy *et al.*, 2012; Chen *et al.*, 2016) with these thresholds: |log_2_ fold-change (FC)| > 1, *p* < 0.05, and false discovery rate (FDR) < 0.05. Subcellular localization predictions were performed using DeepLoc-2.0 (Thumuluri *et al.*, 2022; Sin *et al.*, 2024). Proteins with the highest DeepLoc-2.0 probability scores for nuclear localization are highlighted in orange, with histone proteins specifically annotated.

**Fig. S3.** **Conservation of histone H1 post-translational modifications (PTMs) across five plant species.** Multiple sequence alignments of histone H1 orthologs identified by LC–MS/MS in *Z. mays*, *O. sativa*, *G. max*, *C. annuum*, and *S. lycopersicum* using Clustal Omega (Sievers & Higgins, 2018). Red, methylation sites (+14.01565 Da); magenta, dimethylation sites (+28.03130 Da), cyan, trimethylation sites (+42.04695 Da); yellow, oxidation/hydroxylation sites (+15.99492 Da); green, acetylation sites (+ 42.01057 Da); purple, phosphorylation sites (+79.96633 Da).

**Fig. S4. Conservation of histone H2A post-translational modifications (PTMs) across six plant species**. Multiple sequence alignments of histone H2A orthologs identified by LC–MS/MS in *Z. mays*, *O. sativa*, *G. max*, *C. annuum*, *S. lycopersicum*, and *A. thaliana* using Clustal Omega (Sievers & Higgins, 2018). Red, methylation sites (+14.01565 Da); magenta, dimethylation sites (+28.03130 Da); cyan, trimethylation sites (+42.04695 Da); yellow, oxidation/hydroxylation sites (+15.99492 Da); green, acetylation sites (+ 42.01057 Da); purple, phosphorylation sites (+79.96633 Da).

**Fig. S5.** **Conservation of histone H3 post-translational modifications (PTMs) across six plant species**. Multiple sequence alignments of histone H3 orthologs identified by LC–MS/MS in *Z. mays*, *O. sativa*, *G. max*, *C. annuum*, *S. lycopersicum*, and *A. thaliana* using Clustal Omega (Sievers & Higgins, 2018). Red, methylation sites (+14.01565 Da); magenta, dimethylation sites (+28.03130 Da); cyan, trimethylation sites (+42.04695 Da); yellow, oxidation/hydroxylation sites (+15.99492 Da); green, acetylation sites (+ 42.01057 Da); purple, phosphorylation sites (purple, +79.96633 Da).

**Fig. S6. Site-specific histone H4 post-translational modifications (PTMs) and their dynamic remodeling in response to abiotic stress.** **(a–b).** Representative LC–MS/MS spectra of the tryptic peptides K_Ac_VLRDNIQGITKPAIR **(a)** and TVTAM_Ox_DVVYALKR **(b)** confirming conserved PTMs on histone H4 orthologs in *Z. mays*, *O. sativa*, *G. max*, *C. annuum*, *S. lycopersicum*, and *A. thaliana*. Annotated b- and y-ion series confirmed the peptide sequences and site-localized modifications, with acetylation at H4K20 highlighted in green and oxidation at H4M84 highlighted in yellow. **(c, g)** Relative abundance of K_Ac_VLRDNIQGITKPAIR **(c)** and TVTAM_Ox_DVVYALKR **(g)** in *O. sativa* under control versus salt stress conditions. **(d–j).** Relative abundances of K_Ac_VLRDNIQGITKPAIR **(d–f)** and TVTAM_Ox_DVVYALKR **(h–j)** in *G. max* under salt **(d, h)**, drought **(e, i)**, and heat (**f, j)** stress. Data represent mean ± SE from three biological replicates, determined by label-free quantification and analyzed by one-tailed Student’s t-test. Corresponding MS/MS spectra of the unmodified peptides are provided in **Fig. S7**.

**Fig. S7. Representative MS/MS spectra of selected unmodified peptides from histone H4.** MS/MS spectra of KVLRDNIQGITKPAIR **(a)** and TVTAMDVVYALKR **(b)** in the unmodified peptidoform from *A. thaliana*, *G. max*, *C. annuum*, *S. lycopersicum*, *Z. mays*, and *O. sativa*. Annotated b- and y-ion series confirmed the absence of post-translational modifications on these conserved peptide sequences, serving as controls for the modified peptidoforms presented in **Fig. S6.**

 **Fig. S8. Enriched gene ontology (GO) terms of the differentially expressed proteins (DEPs) in *O. sativa* upon salt treatment.** GO enrichment analyses of down-regulated **(a)** and up-regulated **(b)** DEPs between salt-treated rice and their mock-treated counterparts. GO terms with a fold enrichment > 8 and an adjusted p-value < 0.01 are shown. BP, biological process; MF, molecular function.

 **Fig. S9. Enriched gene ontology (GO) terms of the differentially expressed proteins (DEPs) in *G. max* upon salt treatment.** GO enrichment analyses of down-regulated **(a)** and up-regulated **(b)** DEPs between salt-treated soybean and their mock-treated counterparts. GO terms with a fold enrichment > 8 and an adjusted p-value < 0.01 are shown. BP, biological process; MF, molecular function.

**Fig. S10. Enriched gene ontology (GO) terms of the differentially expressed proteins (DEPs) in *G. max* upon drought treatment.** GO enrichment analyses of down-regulated **(a)** and up-regulated **(b)** DEPs between drought-treated soybean and their mock-treated counterparts. GO terms with a fold enrichment > 8 and an adjusted p-value < 0.01 are shown. BP, biological process; MF, molecular function.

**Fig. S11. Enriched gene ontology (GO) terms of the differentially expressed proteins (DEPs) in *G. max* upon heat treatment.** GO enrichment analyses of down-regulated **(a)** and up-regulated **(b)** DEPs between heat-treated soybean and their mock-treated counterparts. GO terms with a fold enrichment > 8 and an adjusted p-value < 0.01 are shown. BP, biological process; MF, molecular function.

**Fig. S12. Representative MS/MS spectra of selected unmodified peptides from histone H2B.** MS/MS spectra of the unmodified tryptic peptide A/SMSIMNSFINDIFEK of histone H2B orthologs from *Z. mays*, *O. sativa*, *G. max*, *C.annuum*, *S. lycopersicum*, and *A. thaliana*. The annotated b- and y-ion series confirmed the absence of post-translational modifications (PTMs) on this conserved peptide sequence, serving as a control for the PTM sites shown in **Fig. 2**.

**Fig. S13. Combinatorial histone post-translational modifications (PTMs) on conserved histone H2B and H3 peptides.** Representative LC–MS/MS spectra of peptidoforms bearing multiple PTMs from histone orthologs across *Z. mays*, *O. sativa*, *G. max*, *C. annuum,* and *S. lycopersicum.* **(a)** MS/MS spectra of LVLPGELAKHAVSEGTK from histone H2B, showing the co-existing PTMs on distinct amino acids within the same tryptic peptide. **(b)** MS/MS spectra of SQKAGLQFPVGR from histone H3, revealing alternative modification states on the same amino acid residue. Modified amino acids are highlighted in the peptide sequences of the annotated b- and y-ion series: methylation were highlighted in red, dimethylation were highlighted in magenta, oxidation/hydroxylation were highlighted in yellow. These spectra demonstrate the capacity of our LC–MS/MS workflow to resolve complex PTM combinations on individual histone peptides, complementing the single-PTM examples in **Fig. 2** and **Fig. S6**.

**Table S1.** Comparison of conserved histone PTMs with reported PTM patterns in Plant PTM Viewer 2.0.

| *G. max* H2B (A0A0R0HEQ3) |  | |
| --- | --- | --- |
| *C. annuum* H2B (A0A1U8GXA4) |  | |
| *Z. mays* H2B (B6SIF9) |  | |
| *S. lycopersicum* H2B (A0A3Q7FJ91) |  | |
| *G,max* H3 (C6SZA4) |  | |
| *C. annuum* H3 (A0A2G2Y674) |  | |
| *Z. mays* H3 (B6TTP0) |  | |
| *S. lycopersicum* H3 (Q6LB28) |  | |
| *G. max* H4 A0A0R0EX12 |  | |
| *A. thaliana* H4 (P59259) |  |  |

**References:
Chen Y, Lun ATL, Smyth GK**. **2016**. From reads to genes to pathways: differential expression analysis of RNA-Seq experiments using Rsubread and the edgeR quasi-likelihood pipeline. *F1000Research* **5**: 1438.

**Ligges U, Mächler M, Schnackenberg S, Maechler M, Schnackenberg S**. **2003**. Scatterplot3d - an R package for visualizing multivariate data. *J. Stat. Softw* **8**: 1–20.

**McCarthy DJ, Chen Y, Smyth GK**. **2012**. Differential expression analysis of multifactor RNA-Seq experiments with respect to biological variation. *Nucleic Acids Research* **40**: 4288–4297.

**Robinson MD, McCarthy DJ, Smyth GK**. **2009**. edgeR: A Bioconductor package for differential expression analysis of digital gene expression data. *Bioinformatics* **26**: 139–140.

**Sievers F, Higgins DG**. **2018**. Clustal Omega for making accurate alignments of many protein sequences. *Protein Science* **27**: 135–145.

**Sin WC, Liu J, Zhong JY, Lam HM, Lim BL**. **2024**. Comparative proteomics analysis of root and nodule mitochondria of soybean. *Plant Cell Environ* **49**, 3482–3499.

**Thumuluri V, Armenteros, Juan Almagro, Johansen AR, Nielsen H, Winther O**. **2022**. DeepLoc 2.0: multi-label subcellular localization prediction using protein language models. *Nucleic Acids Research* **50**: W228-W234.
